# Comparison of Relationships Between the Number of Deaths Due to the 10 Leading Causes and Air Temperature in Hokkaido and Okinawa, Japan

**DOI:** 10.7759/cureus.68291

**Published:** 2024-08-31

**Authors:** Hiroe Kitamura, Hiromi Suzuki, Yoshiro Mori, Masaki Bando, Yukio Yamamoto, Yasuko Maekawa, Nobuyuki Miyatake

**Affiliations:** 1 Department of Hygiene, Faculty of Medicine, Kagawa University, Miki, JPN; 2 Department of Nursing, Shikoku Medical College, Utazu, JPN; 3 Department of Public Health, Graduate School of Biomedical Sciences, Tokushima University, Tokushima, JPN; 4 Department of Physical Therapy, Kenshokai College of Health and Welfare, Tokushima, JPN; 5 Department of Judo Therapy, Shikoku Medical College, Utazu, JPN; 6 Faculty of Nursing, Kansai University of Social Welfare, Ako, JPN

**Keywords:** ecological study, okinawa, hokkaido, air temperature, number of deaths

## Abstract

Background

Some literature reports an association between air temperature and mortality in certain diseases. However, the relationships between air temperature parameters and all causes of death have not been thoroughly explored in Japan.

Objective

This study examined the relationships between the number of deaths from the 10 leading causes and air temperature parameters in Hokkaido (the northernmost region) and Okinawa (the southernmost region) prefectures in Japan.

Methods

We collected monthly data on the number of deaths from the 10 leading causes and air temperature parameters in Hokkaido and Okinawa prefectures from January 2008 to December 2022 using information from official sources. Annual population data for each prefecture were also obtained. The relationships between the number of deaths and air temperature parameters were assessed through an ecological study.

Results

The mean air temperature was 9.59 ± 9.23 °C in Hokkaido and 23.46 ± 4.37 °C in Okinawa, with all temperature parameters significantly lower in Hokkaido than in Okinawa. The number of deaths from the 10 leading causes, excluding aspiration pneumonia, was significantly higher in Hokkaido for both sexes compared to Okinawa. In Hokkaido, deaths due to heart disease, cerebrovascular disease, pneumonia, accidents, and renal failure showed a significant correlation with all air temperature parameters for both sexes. In Okinawa, heart disease and cerebrovascular disease deaths were correlated with all air temperature parameters for both sexes.

Conclusions

The relationships between the number of deaths from the 10 leading causes and air temperature parameters differed between Hokkaido and Okinawa prefectures in Japan.

## Introduction

Air temperature has been linked to various health issues both in Japan and globally [1-3]. Elevated temperatures are associated with heat stroke [4], while lower temperatures have been connected to heart disease [5], cerebrovascular disease [6], and pneumonia [7].

In our previous research, we explored the impact of air temperature on health outcomes. We found that higher temperatures were associated with increased ambulance transports in Japan [8]. Conversely, lower temperatures were related to more ambulance transports and deaths in Takamatsu City, Japan [9]. Data from the 23 wards of Tokyo indicated a connection between lower temperatures and deaths from accidents such as asphyxia [10], drowning [11], and falls [12]. Additionally, a quadratic model showed that the relationship between air temperature and mortality was consistent across all 47 prefectures in Japan [13,14]. These findings suggest that both high and low temperatures are linked to health outcomes. However, the impact of air temperature on renal failure mortality and other major causes of death shows regional differences between Hokkaido and Okinawa, highlighting the need for further investigation [15].

To address these gaps, we conducted a study using data from January 2008 to December 2022, extending beyond the previous study's timeframe [15]. We examined the relationships between mortality from the 10 leading causes of death and air temperature parameters in Hokkaido and Okinawa. This research focused on population, community, and ecosystem levels, analyzing mortality data and environmental factors, including air temperature, through a descriptive and cross-sectional ecological approach.

## Materials and methods

Study area

In this ecological study, we selected Hokkaido and Okinawa prefectures to represent the extremes of Japan’s climate. Hokkaido, situated in the northernmost part of Japan (43°03'51"N), has a population of 5,224,614, spans an area of 83,424 km², and has a population density of 66.6 inhabitants per km². In contrast, Okinawa, located in the southernmost part of Japan (26°12'14"N), has a population of 1,467,480, covers an area of 2,281 km², and has a population density of 642.9 inhabitants per km² [16,17].

Ten leading causes of death

In Japan, the 10 leading causes of death for 2022 included malignant neoplasms, heart disease, senility, cerebrovascular disease, pneumonia, aspiration pneumonia, accidents, renal failure, Alzheimer’s disease, and vascular and unspecified dementia [18]. These rankings are based on the most recent official data, which may fluctuate slightly from year to year. Aspiration pneumonia, a new category added in 2017, was included in our study from January 2017 to December 2022. Data on deaths from these 10 causes for the period between January 2008 and December 2022 were sourced from official websites of Hokkaido and Okinawa prefectures [19,20]. Additionally, we obtained annual population data for these prefectures from official sources [21] and adjusted the death counts for population size and the number of days in each month.

Air temperature parameters

Monthly air temperature data from January 2008 to December 2022 were obtained from the Japan Meteorological Agency [22]. The parameters collected included mean air temperature (°C), mean of the highest air temperature (°C), mean of the lowest air temperature (°C), highest air temperature (°C), and lowest air temperature (°C).

Statistical analysis

Data were presented as mean ± SD. Simple correlation analysis was used to examine the relationships between the number of deaths due to the 10 leading causes and air temperature parameters. Additionally, the Kruskal-Wallis test and Steel test were employed to compare the number of deaths stratified by month, with a significance level set at p < 0.05. All statistical analyses were performed using JMP Pro 17 (SAS Institute Inc., NC, USA).

Ethics

All data for this study were sourced from official home pages. Ethical approval was granted by the Ethical Committee of Shikoku Medical College, Utazu, Japan (approval number R5-03-002).

## Results

Table 1 presents the number of deaths from the 10 leading causes and the associated air temperature parameters in Hokkaido and Okinawa prefectures.

**Table 1:** Clinical data on the number of deaths due to the 10 leading causes and air temperature parameters in Hokkaido and Okinawa, Japan Number of deaths due to the 10 leading causes: per 100,000 people/month; number of months = 180; number of months = 72 (aspiration pneumonia)

	Hokkaido					Okinawa					p
	Mean	±	SD	Minimum	Maximum	Mean	±	SD	Minimum	Maximum	
Malignant neoplasms (Total)	29.03	±	2.38	23.49	34.2	17.49	±	1.57	14.29	23.42378	<0.01
Malignant neoplasms (Men)	16.8	±	1.14	13.72	19.87	10.33	±	1.07	8	14.94	<0.01
Malignant neoplasms (Women)	12.23	±	1.36	9.29	15.35	7.15	±	0.87	5.29	9.96	<0.01
Cardiac disease (Total)	14.52	±	1.86	11.05	21.25	9.3	±	1.31	6.43	13.46	<0.01
Cardiac disease (Men)	6.72	±	0.97	4.9	9.68	4.65	±	0.85	2.97	7.53	<0.01
Cardiac disease (Women)	7.79	±	0.98	5.89	11.57	4.65	±	0.74	2.94	6.76	<0.01
Senility (Total)	4.9	±	2.77	1.23	13.52	4.02	±	2.42	0.85	13.05	<0.01
Senility (Men)	1.25	±	0.75	0.19	3.81	1.03	±	0.75	0.07	4.03	<0.01
Senility (Women)	3.64	±	2.03	0.93	9.7	3.01	±	1.69	0.57	9.01	<0.01
Heart disease (Total)	7.71	±	0.73	5.71	9.87	5.32	±	0.75	2.85	7.28	<0.01
Heart disease (Men)	3.77	±	0.45	2.64	5.07	2.8	±	0.51	1.11	3.86	<0.01
Heart disease (Women)	3.95	±	0.4	2.67	4.93	2.52	±	0.45	1.26	3.74	<0.01
Pneumonia (Total)	7.5	±	1.46	4.21	11.54	4.53	±	1.33	1.21	7.35	<0.01
Pneumonia (Men)	4.18	±	0.82	2.37	6.69	2.47	±	0.7	0.74	3.96	<0.01
Pneumonia (Women)	3.31	±	0.69	1.81	5.06	2.05	±	0.76	0.33	3.89	<0.01
Aspiration pneumonia (Total)	2.21	±	0.56	1.22	3.66	2.19	±	0.4	1.49	2.96	0.8
Aspiration pneumonia (Men)	1.26	±	0.32	0.7	2.17	1.27	±	0.34	0.54	2.08	0.85
Aspiration pneumonia (Women)	0.95	±	0.26	0.46	1.65	0.92	±	0.24	0.53	1.69	0.47
Accidents (Total)	2.57	±	0.47	1.68	4.34	1.68	±	0.44	0.75	3.03	<0.01
Accidents (Men)	1.5	±	0.26	1.06	2.35	1.11	±	0.33	0.47	2.34	<0.01
Accidents (Women)	1.07	±	0.26	0.56	1.98	0.56	±	0.21	0.13	1.07	<0.01
Renal failure (Total)	2.4	±	0.38	1.65	3.74	1.29	±	0.37	0.5	2.42	<0.01
Renal failure (Men)	1.16	±	0.23	0.77	2.08	0.58	±	0.23	0.13	1.28	<0.01
Renal failure (Women)	1.23	±	0.2	0.8	1.89	0.74	±	0.24	0.27	1.72	<0.01
Alzheimer’s disease (Total)	0.9	±	0.65	0.1	2.46	0.41	±	0.37	0	1.81	<0.01
Alzheimer’s disease (Men)	0.32	±	0.23	0.03	0.9	0.15	±	0.15	0	0.67	<0.01
Alzheimer’s disease (Women)	0.58	±	0.43	0.05	1.62	0.26	±	0.24	0	1.14	<0.01
Vascular and unspecified dementia (Total)	1.06	±	0.64	0.12	2.43	0.5	±	0.33	0	1.75	<0.01
Vascular and unspecified dementia (Men)	0.37	±	0.26	0.03	1.25	0.17	±	0.16	0	0.74	<0.01
Vascular and unspecified dementia (Women)	0.69	±	0.39	0.08	1.54	0.33	±	0.22	0	1.21	<0.01
Mean air temperature (°C)	9.59	±	9.23	-4.7	24.8	23.46	±	4.37	14.9	30.4	<0.01
Mean of the highest air temperature (°C)	13.53	±	9.84	-2	29.1	26.14	±	4.45	17	33.6	<0.01
Mean of the lowest air temperature (°C)	6.09	±	9.12	-8.2	21.3	21.27	±	4.5	12.7	28.3	<0.01
The highest air temperature (°C)	20.85	±	9.86	1.8	35.1	29.43	±	3.41	22.6	35.1	<0.01
The lowest air temperature (°C)	0.86	±	9.36	-14.9	16.9	17.59	±	5.24	6.1	26.4	<0.01

The number of deaths due to the 10 leading causes (excluding aspiration pneumonia) was significantly higher in Hokkaido prefecture compared to Okinawa prefecture. The mean air temperature in Hokkaido was 9.59 ± 9.23 °C, while in Okinawa it was 23.46 ± 4.37 °C. All air temperature parameters were significantly lower in Hokkaido than in Okinawa.

We subsequently analyzed the relationship between the number of deaths from the 10 leading causes and air temperature parameters in each prefecture, as detailed in Table 2.

**Table 2:** Relationships between the number of deaths due to the 10 leading causes of death and air temperature parameters in Hokkaido and Okinawa, Japan Number of deaths due to the 10 leading causes: per 100,000 people/day; number of months = 180; number of months = 72 (aspiration pneumonia)

		Hokkaido						Okinawa					
		Total		Men		Women		Total		Men		Women	
		r	p	r	p	r	p	r	p	r	p	r	p
Malignant neoplasms	Mean air temperature (°C)	-0.03	0.65	-0.07	0.32	0	0.99	-0.03	0.65	-0.03	0.69	-0.02	0.74
	Mean of the highest air temperature (°C)	-0.04	0.62	-0.08	0.3	0	0.98	-0.04	0.61	-0.03	0.67	-0.03	0.71
	Mean of the lowest air temperature (°C)	-0.03	0.65	-0.08	0.31	0	0.98	-0.03	0.66	-0.03	0.69	-0.02	0.77
	The highest air temperature (°C)	-0.04	0.64	-0.08	0.3	0	0.99	-0.05	0.53	-0.04	0.57	-0.03	0.67
	The lowest air temperature (°C)	-0.04	0.63	-0.08	0.28	0	0.97	-0.02	0.83	-0.02	0.79	0	0.96
Heart disease	Mean air temperature (°C)	-0.74	<0.01	-0.78	<0.01	-0.62	<0.01	-0.42	<0.01	-0.34	<0.01	-0.35	<0.01
	Mean of the highest air temperature (°C)	-0.74	<0.01	-0.78	<0.01	-0.63	<0.01	-0.42	<0.01	-0.34	<0.01	-0.34	<0.01
	Mean of the lowest air temperature (°C)	-0.74	<0.01	-0.78	<0.01	-0.62	<0.01	-0.42	<0.01	-0.34	<0.01	-0.35	<0.01
	The highest air temperature (°C)	-0.73	<0.01	-0.78	<0.01	-0.61	<0.01	-0.43	<0.01	-0.35	<0.01	-0.36	<0.01
	The lowest air temperature (°C)	-0.73	<0.01	-0.77	<0.01	-0.61	<0.01	-0.39	<0.01	-0.32	<0.01	-0.32	<0.01
Senility	Mean air temperature (°C)	-0.05	0.53	-0.04	0.56	-0.05	0.53	0.02	0.82	0.03	0.74	0.01	0.86
	Mean of the highest air temperature (°C)	-0.04	0.55	-0.04	0.58	-0.05	0.55	0	0.97	0.01	0.89	0	1
	Mean of the lowest air temperature (°C)	-0.05	0.51	-0.05	0.53	-0.05	0.51	0.02	0.76	0.03	0.69	0.02	0.8
	The highest air temperature (°C)	-0.03	0.68	-0.03	0.67	-0.03	0.69	-0.01	0.92	0	0.98	-0.01	0.89
	The lowest air temperature (°C)	-0.05	0.47	-0.05	0.47	-0.05	0.48	0.03	0.73	0.04	0.62	0.02	0.78
Cerebrovascular disease	Mean air temperature (°C)	-0.72	<0.01	-0.67	<0.01	-0.56	<0.01	-0.44	<0.01	-0.45	<0.01	-0.23	0
	Mean of the highest air temperature (°C)	-0.72	<0.01	-0.67	<0.01	-0.56	<0.01	-0.44	<0.01	-0.45	<0.01	-0.23	0
	Mean of the lowest air temperature (°C)	-0.72	<0.01	-0.66	<0.01	-0.56	<0.01	-0.45	<0.01	-0.45	<0.01	-0.24	0
	The highest air temperature (°C)	-0.73	<0.01	-0.67	<0.01	-0.58	<0.01	-0.43	<0.01	-0.46	<0.01	-0.2	0.01
	The lowest air temperature (°C)	-0.71	<0.01	-0.65	<0.01	-0.56	<0.01	-0.45	<0.01	-0.45	<0.01	-0.24	0
Pneumonia	Mean air temperature (°C)	-0.38	<0.01	-0.42	<0.01	-0.3	<0.01	-0.23	<0.01	-0.32	<0.01	-0.1	0.16
	Mean of the highest air temperature (°C)	-0.38	<0.01	-0.42	<0.01	-0.31	<0.01	-0.22	<0.01	-0.31	<0.01	-0.09	0.21
	Mean of the lowest air temperature (°C)	-0.37	<0.01	-0.41	<0.01	-0.3	<0.01	-0.24	<0.01	-0.33	<0.01	-0.11	0.15
	The highest air temperature (°C)	-0.39	<0.01	-0.43	<0.01	-0.31	<0.01	-0.22	<0.01	-0.31	<0.01	-0.1	0.17
	The lowest air temperature (°C)	-0.37	<0.01	-0.42	<0.01	-0.29	<0.01	-0.22	<0.01	-0.31	<0.01	-0.1	0.18
Aspiration pneumonia	Mean air temperature (°C)	-0.04	0.73	-0.01	0.95	-0.08	0.51	-0.1	0.41	-0.12	0.33	0	1
	Mean of the highest air temperature (°C)	-0.05	0.67	-0.02	0.88	-0.08	0.48	-0.1	0.41	-0.12	0.3	0.01	0.93
	Mean of the lowest air temperature (°C)	-0.04	0.74	0	0.99	-0.08	0.5	-0.1	0.41	-0.12	0.33	0	0.99
	The highest air temperature (°C)	-0.08	0.53	-0.04	0.75	-0.11	0.35	-0.13	0.26	-0.12	0.31	-0.05	0.65
	The lowest air temperature (°C)	-0.05	0.69	-0.02	0.88	-0.08	0.51	-0.08	0.5	-0.08	0.49	-0.02	0.88
Accidents	Mean air temperature (°C)	-0.61	<0.01	-0.58	<0.01	-0.52	<0.01	-0.12	0.11	-0.09	0.24	-0.11	0.14
	Mean of the highest air temperature (°C)	-0.61	<0.01	-0.58	<0.01	-0.52	<0.01	-0.11	0.14	-0.08	0.3	-0.1	0.16
	Mean of the lowest air temperature (°C)	-0.61	<0.01	-0.58	<0.01	-0.52	<0.01	-0.13	0.08	-0.1	0.19	-0.12	0.12
	The highest air temperature (°C)	-0.61	<0.01	-0.58	<0.01	-0.51	<0.01	-0.11	0.14	-0.07	0.35	-0.12	0.11
	The lowest air temperature (°C)	-0.61	<0.01	-0.58	<0.01	-0.52	<0.01	-0.11	0.15	-0.07	0.32	-0.1	0.16
Renal failure	Mean air temperature (°C)	-0.45	<0.01	-0.31	<0.01	-0.47	<0.01	-0.19	0.01	-0.16	0.04	-0.16	0.03
	Mean of the highest air temperature (°C)	-0.45	<0.01	-0.31	<0.01	-0.47	<0.01	-0.19	0.01	-0.15	0.04	-0.16	0.04
	Mean of the lowest air temperature (°C)	-0.45	<0.01	-0.31	<0.01	-0.47	<0.01	-0.2	0.01	-0.16	0.04	-0.16	0.03
	The highest air temperature (°C)	-0.45	<0.01	-0.32	<0.01	-0.47	<0.01	-0.22	0	-0.16	0.03	-0.19	0.01
	The lowest air temperature (°C)	-0.44	<0.01	-0.3	<0.01	-0.48	<0.01	-0.17	0.02	-0.14	0.06	-0.14	0.06
Alzheimer’s disease	Mean air temperature (°C)	0.02	0.79	0.05	0.51	0	0.96	0.01	0.9	-0.07	0.35	0.06	0.43
	Mean of the highest air temperature (°C)	0.02	0.76	0.05	0.48	0.01	0.94	0	0.98	-0.08	0.28	0.05	0.52
	Mean of the lowest air temperature (°C)	0.02	0.82	0.05	0.55	0	0.99	0.02	0.84	-0.06	0.39	0.06	0.39
	The highest air temperature (°C)	0.04	0.62	0.07	0.36	0.02	0.8	-0.02	0.78	-0.09	0.21	0.03	0.71
	The lowest air temperature (°C)	0.01	0.87	0.04	0.56	0	0.95	0.01	0.88	-0.06	0.42	0.06	0.46
Vascular and unspecified dementia	Mean air temperature (°C)	-0.04	0.56	-0.01	0.9	-0.07	0.38	0	0.96	-0.08	0.3	0.05	0.5
	Mean of the highest air temperature (°C)	-0.04	0.57	-0.01	0.91	-0.06	0.39	-0.01	0.89	-0.09	0.25	0.05	0.52
	Mean of the lowest air temperature (°C)	-0.05	0.53	-0.01	0.88	-0.07	0.35	0	0.98	-0.07	0.33	0.05	0.51
	The highest air temperature (°C)	-0.03	0.73	0.01	0.92	-0.05	0.52	-0.01	0.85	-0.09	0.21	0.05	0.53
	The lowest air temperature (°C)	-0.05	0.5	-0.01	0.87	-0.07	0.32	0	0.96	-0.08	0.3	0.05	0.5

In Hokkaido prefecture, the number of deaths from heart disease, cerebrovascular disease, pneumonia, accidents, and renal failure showed a correlation with all air temperature parameters for both sexes. In Okinawa prefecture, the number of deaths due to heart disease and cerebrovascular disease correlated with all air temperature parameters. Additionally, deaths from pneumonia correlated with all air temperature parameters in men but not in women. The number of deaths due to renal failure correlated with all air temperature parameters, except for the lowest air temperature, in both sexes.

We next compared the number of deaths due to the 10 leading causes of death across different months (Table 3).

**Table 3:** Comparison of monthly deaths due to the 10 leading causes of death in Hokkaido and Okinawa, Japan a: p = 0.05 vs. January; per 100,000 people/day. b: p = 0.05 vs. February; per 100,000 people/day Number of months = 180; number of months = 72 (aspiration pneumonia)

	Month	Hokkaido												Okinawa											
		Total				Men				Women				Total				Men				Women			
Malignant neoplasms	January	0.97	±	0.08		0.56	±	0.04		0.41	±	0.04		0.57	±	0.04		0.34	±	0.03		0.23	±	0.03	
	February	0.95	±	0.08		0.55	±	0.04		0.4	±	0.04		0.59	±	0.05		0.35	±	0.03		0.24	±	0.04	
	March	0.95	±	0.08		0.54	±	0.03		0.4	±	0.05		0.57	±	0.04		0.33	±	0.03		0.24	±	0.03	
	April	0.94	±	0.07		0.54	±	0.04		0.39	±	0.04		0.57	±	0.06		0.34	±	0.03		0.23	±	0.03	
	May	0.94	±	0.08		0.55	±	0.03		0.39	±	0.05		0.56	±	0.04		0.34	±	0.03		0.22	±	0.02	
	June	0.94	±	0.08		0.54	±	0.03		0.4	±	0.05		0.57	±	0.06		0.34	±	0.04		0.23	±	0.03	
	July	0.94	±	0.07		0.54	±	0.03		0.4	±	0.05		0.56	±	0.06		0.34	±	0.04		0.23	±	0.03	
	August	0.95	±	0.07		0.55	±	0.04		0.4	±	0.04		0.58	±	0.07		0.34	±	0.05		0.24	±	0.03	
	September	0.96	±	0.08		0.56	±	0.04		0.41	±	0.05		0.57	±	0.04		0.34	±	0.03		0.23	±	0.03	
	October	0.97	±	0.07		0.57	±	0.04		0.4	±	0.04		0.59	±	0.04		0.34	±	0.03		0.25	±	0.02	
	November	0.97	±	0.07		0.57	±	0.03		0.41	±	0.04		0.57	±	0.05		0.34	±	0.03		0.23	±	0.04	
	December	0.96	±	0.07		0.56	±	0.03		0.4	±	0.04		0.6	±	0.04		0.36	±	0.03		0.24	±	0.02	
Heart disease	January	0.57	±	0.05		0.27	±	0.02		0.3	±	0.03		0.35	±	0.04		0.18	±	0.03		0.17	±	0.03	
	February	0.52	±	0.03	a	0.25	±	0.02	a	0.27	±	0.02		0.35	±	0.03		0.18	±	0.02		0.17	±	0.03	
	March	0.5	±	0.03	a	0.23	±	0.02	a	0.27	±	0.02		0.33	±	0.05		0.16	±	0.03		0.17	±	0.03	
	April	0.47	±	0.03	a	0.22	±	0.01	a	0.25	±	0.02	a	0.3	±	0.05	b	0.15	±	0.03	b	0.15	±	0.02	
	May	0.46	±	0.03	a	0.21	±	0.01	a	0.25	±	0.03	a	0.29	±	0.02	b	0.15	±	0.02	b	0.14	±	0.02	a
	June	0.43	±	0.03	a	0.2	±	0.02	a	0.23	±	0.02	a	0.28	±	0.03	b	0.14	±	0.02	b	0.14	±	0.02	a
	July	0.43	±	0.04	a	0.19	±	0.02	a	0.23	±	0.02	a	0.3	±	0.03	b	0.16	±	0.03		0.15	±	0.02	
	August	0.42	±	0.04	a	0.19	±	0.02	a	0.23	±	0.02	a	0.3	±	0.05	b	0.15	±	0.03	b	0.15	±	0.02	
	September	0.42	±	0.03	a	0.19	±	0.02	a	0.23	±	0.02	a	0.29	±	0.03	b	0.14	±	0.02	b	0.15	±	0.02	
	October	0.46	±	0.04	a	0.21	±	0.02	a	0.25	±	0.02	a	0.28	±	0.04	b	0.13	±	0.02	b	0.14	±	0.02	a
	November	0.49	±	0.05	a	0.23	±	0.02	a	0.26	±	0.03	a	0.29	±	0.04	b	0.15	±	0.03	b	0.14	±	0.02	a
	December	0.54	±	0.05	a	0.26	±	0.02	a	0.28	±	0.03		0.32	±	0.03		0.16	±	0.02		0.16	±	0.02	
Senility	January	0.16	±	0.1		0.04	±	0.02		0.12	±	0.07		0.13	±	0.07		0.03	±	0.02		0.1	±	0.05	
	February	0.16	±	0.09		0.04	±	0.02		0.12	±	0.06		0.14	±	0.07		0.03	±	0.02		0.1	±	0.05	
	March	0.16	±	0.09		0.04	±	0.02		0.12	±	0.06		0.13	±	0.07		0.03	±	0.02		0.09	±	0.04	
	April	0.17	±	0.09		0.04	±	0.02		0.12	±	0.06		0.12	±	0.07		0.03	±	0.02		0.09	±	0.05	
	May	0.15	±	0.09		0.04	±	0.02		0.11	±	0.06		0.12	±	0.07		0.03	±	0.02		0.09	±	0.05	
	June	0.15	±	0.08		0.04	±	0.02		0.11	±	0.06		0.12	±	0.08		0.03	±	0.02		0.09	±	0.06	
	July	0.14	±	0.08		0.04	±	0.02		0.11	±	0.06		0.13	±	0.08		0.03	±	0.02		0.09	±	0.06	
	August	0.15	±	0.09		0.04	±	0.02		0.11	±	0.06		0.13	±	0.08		0.03	±	0.03		0.09	±	0.05	
	September	0.16	±	0.09		0.04	±	0.03		0.12	±	0.07		0.15	±	0.09		0.04	±	0.03		0.11	±	0.07	
	October	0.18	±	0.1		0.05	±	0.03		0.13	±	0.08		0.14	±	0.09		0.04	±	0.03		0.1	±	0.07	
	November	0.18	±	0.1		0.05	±	0.03		0.14	±	0.08		0.14	±	0.09		0.04	±	0.03		0.1	±	0.07	
	December	0.18	±	0.11		0.05	±	0.03		0.13	±	0.08		0.15	±	0.1		0.04	±	0.03		0.11	±	0.07	
Cerebrovascular disease	January	0.29	±	0.02		0.15	±	0.01		0.14	±	0.01		0.2	±	0.02		0.11	±	0.01		0.09	±	0.02	
	February	0.28	±	0.02	a	0.14	±	0.01	a	0.14	±	0.01		0.19	±	0.02		0.1	±	0.02		0.09	±	0.02	
	March	0.26	±	0.02	a	0.13	±	0.01	a	0.13	±	0.01		0.19	±	0.02		0.1	±	0.01		0.09	±	0.01	
	April	0.25	±	0.02	a	0.12	±	0.01	a	0.12	±	0.01	a	0.17	±	0.02	a	0.09	±	0.01	a	0.08	±	0.02	
	May	0.24	±	0.02	a	0.12	±	0.01	a	0.12	±	0.01	a	0.17	±	0.03		0.09	±	0.02		0.08	±	0.02	
	June	0.24	±	0.02	a	0.11	±	0.01	a	0.12	±	0.01	a	0.17	±	0.03	a	0.09	±	0.02		0.08	±	0.01	
	July	0.23	±	0.01	a	0.11	±	0.01	a	0.12	±	0.01	a	0.16	±	0.03	a	0.08	±	0.02	a	0.08	±	0.01	
	August	0.23	±	0.01	a	0.11	±	0.01	a	0.12	±	0.01	a	0.17	±	0.02	a	0.09	±	0.01	a	0.08	±	0.02	
	September	0.23	±	0.02	a	0.11	±	0.01	a	0.12	±	0.01	a	0.16	±	0.02	a	0.08	±	0.01	a	0.08	±	0.01	
	October	0.26	±	0.01	a	0.12	±	0.01	a	0.13	±	0.01		0.16	±	0.03	a	0.08	±	0.01	a	0.08	±	0.02	
	November	0.27	±	0.02	a	0.13	±	0.01	a	0.13	±	0.01		0.18	±	0.02		0.1	±	0.01		0.08	±	0.01	
	December	0.27	±	0.02	a	0.13	±	0.01	a	0.14	±	0.01		0.19	±	0.01		0.1	±	0.01		0.09	±	0.01	
Pneumonia	January	0.3	±	0.05		0.13	±	0.03		0.13	±	0.03		0.17	±	0.04		0.09	±	0.02		0.08	±	0.03	
	February	0.28	±	0.05		0.12	±	0.03		0.12	±	0.03		0.18	±	0.05		0.1	±	0.03		0.08	±	0.02	
	March	0.25	±	0.04		0.11	±	0.02		0.11	±	0.02		0.16	±	0.04		0.1	±	0.02		0.07	±	0.03	
	April	0.24	±	0.04	a	0.11	±	0.03	a	0.11	±	0.02		0.15	±	0.04		0.08	±	0.02		0.07	±	0.02	
	May	0.24	±	0.05	a	0.1	±	0.02	a	0.1	±	0.02		0.15	±	0.06		0.08	±	0.03		0.07	±	0.03	
	June	0.22	±	0.05	a	0.1	±	0.03	a	0.1	±	0.02	a	0.13	±	0.04		0.07	±	0.02	b	0.06	±	0.03	
	July	0.22	±	0.04	a	0.1	±	0.02	a	0.1	±	0.02	a	0.15	±	0.04		0.08	±	0.02		0.07	±	0.02	
	August	0.23	±	0.05	a	0.1	±	0.02	a	0.1	±	0.02	a	0.15	±	0.04		0.08	±	0.02		0.08	±	0.03	
	September	0.24	±	0.04	a	0.1	±	0.02	a	0.1	±	0.02		0.15	±	0.03		0.08	±	0.02		0.06	±	0.02	
	October	0.25	±	0.04		0.11	±	0.03		0.11	±	0.02		0.14	±	0.03		0.07	±	0.02		0.06	±	0.02	
	November	0.26	±	0.04		0.11	±	0.02		0.11	±	0.02		0.13	±	0.04		0.07	±	0.02		0.06	±	0.02	
	December	0.25	±	0.05		0.11	±	0.03		0.11	±	0.02		0.14	±	0.04		0.08	±	0.02		0.06	±	0.02	
Aspiration pneumonia	January	0.08	±	0.02		0.04	±	0.01		0.04	±	0.01		0.08	±	0.01		0.04	±	0.01		0.03	±	0.01	
	February	0.07	±	0.02		0.04	±	0.01		0.03	±	0.01		0.08	±	0.02		0.05	±	0.01		0.03	±	0.01	
	March	0.07	±	0.02		0.04	±	0.01		0.03	±	0.01		0.07	±	0.02		0.04	±	0.02		0.03	±	0.01	
	April	0.07	±	0.01		0.04	±	0.01		0.03	±	0.01		0.07	±	0.01		0.04	±	0.01		0.03	±	0.01	
	May	0.07	±	0.02		0.04	±	0.01		0.03	±	0.01		0.07	±	0.01		0.03	±	0.02		0.04	±	0.01	
	June	0.07	±	0.02		0.04	±	0.01		0.03	±	0.01		0.07	±	0.01		0.04	±	0.01		0.03	±	0.01	
	July	0.07	±	0.01		0.04	±	0.01		0.03	±	0.01		0.07	±	0.01		0.04	±	0.01		0.03	±	0.01	
	August	0.08	±	0.02		0.05	±	0.02		0.03	±	0.01		0.08	±	0.01		0.05	±	0.01		0.04	±	0.01	
	September	0.08	±	0.02		0.04	±	0.01		0.04	±	0.01		0.07	±	0.01		0.04	±	0.01		0.03	±	0	
	October	0.07	±	0.02		0.04	±	0.01		0.03	±	0.01		0.07	±	0.02		0.04	±	0.01		0.03	±	0.01	
	November	0.08	±	0.02		0.05	±	0.01		0.03	±	0.01		0.07	±	0.01		0.04	±	0.01		0.03	±	0	
	December	0.08	±	0.02		0.04	±	0.01		0.04	±	0.01		0.07	±	0.02		0.05	±	0.01		0.03	±	0.01	
Accidents	January	0.1	±	0.02		0.06	±	0.01		0.04	±	0.01		0.07	±	0.02		0.04	±	0.01		0.02	±	0.01	
	February	0.09	±	0.02		0.05	±	0.01		0.04	±	0.01		0.06	±	0.01		0.04	±	0.01		0.02	±	0.01	
	March	0.08	±	0.01	a	0.05	±	0.01	a	0.03	±	0.01		0.06	±	0.01		0.04	±	0.01		0.02	±	0	
	April	0.08	±	0.01	a	0.05	±	0.01	a	0.03	±	0.01	a	0.05	±	0.01	a	0.03	±	0.01	a	0.02	±	0.01	
	May	0.08	±	0.01	a	0.05	±	0	a	0.03	±	0.01		0.05	±	0.01		0.03	±	0.01		0.02	±	0.01	
	June	0.07	±	0.01	a	0.04	±	0	a	0.03	±	0	a	0.05	±	0.01	a	0.03	±	0.01	a	0.02	±	0.01	
	July	0.07	±	0.01	a	0.04	±	0.01	a	0.03	±	0.01	a	0.06	±	0.01		0.04	±	0.01		0.02	±	0.01	
	August	0.08	±	0.01	a	0.04	±	0.01	a	0.03	±	0.01	a	0.06	±	0.01		0.04	±	0.01		0.02	±	0.01	
	September	0.07	±	0.01	a	0.04	±	0	a	0.03	±	0.01	a	0.06	±	0.01		0.04	±	0.01		0.02	±	0.01	
	October	0.08	±	0.01	a	0.05	±	0.01		0.03	±	0.01	a	0.05	±	0.01		0.03	±	0.01		0.02	±	0.01	
	November	0.09	±	0.01		0.05	±	0.01		0.04	±	0.01		0.04	±	0.01	a	0.03	±	0.01	a	0.02	±	0.01	
	December	0.1	±	0.02		0.06	±	0.01		0.04	±	0.01		0.06	±	0.01		0.04	±	0.01		0.02	±	0.01	
Renal failure	January	0.09	±	0.01		0.04	±	0.01		0.05	±	0.01		0.05	±	0.01		0.02	±	0.01		0.02	±	0.01	
	February	0.08	±	0.01		0.04	±	0.01		0.04	±	0.01		0.05	±	0.01		0.02	±	0.01		0.03	±	0.01	
	March	0.08	±	0.01		0.04	±	0.01		0.04	±	0.01		0.05	±	0.01		0.02	±	0.01		0.03	±	0.01	
	April	0.08	±	0.01		0.04	±	0.01		0.04	±	0.01		0.04	±	0.01		0.02	±	0.01		0.02	±	0.01	
	May	0.08	±	0.01	a	0.04	±	0.01		0.04	±	0.01	a	0.04	±	0.01		0.02	±	0.01		0.02	±	0.01	
	June	0.07	±	0.01	a	0.04	±	0.01		0.04	±	0.01	a	0.04	±	0.01		0.02	±	0.01		0.02	±	0.01	
	July	0.07	±	0.01	a	0.03	±	0.01		0.04	±	0	a	0.04	±	0.01		0.02	±	0.01		0.02	±	0.01	
	August	0.07	±	0.01	a	0.04	±	0.01		0.04	±	0.01	a	0.04	±	0.01		0.02	±	0.01		0.02	±	0.01	
	September	0.07	±	0.01	a	0.03	±	0.01	a	0.04	±	0.01		0.04	±	0.01		0.02	±	0.01		0.02	±	0.01	
	October	0.08	±	0.01		0.04	±	0.01		0.04	±	0.01		0.04	±	0.01		0.02	±	0.01		0.02	±	0.01	
	November	0.09	±	0.02		0.04	±	0.01		0.04	±	0.01		0.04	±	0.01		0.02	±	0		0.02	±	0.01	
	December	0.09	±	0.01		0.04	±	0.01		0.04	±	0.01		0.04	±	0.01		0.02	±	0.01		0.02	±	0.01	
Alzheimer's disease	January	0.03	±	0.02		0.01	±	0.01		0.02	±	0.01		0.02	±	0.02		0.01	±	0.01		0.01	±	0.01	
	February	0.03	±	0.02		0.01	±	0.01		0.02	±	0.01		0.01	±	0.01		0.01	±	0		0.01	±	0.01	
	March	0.03	±	0.02		0.01	±	0.01		0.02	±	0.01		0.01	±	0.01		0	±	0		0.01	±	0.01	
	April	0.03	±	0.02		0.01	±	0.01		0.02	±	0.01		0.01	±	0.01		0	±	0		0.01	±	0.01	
	May	0.03	±	0.02		0.01	±	0.01		0.02	±	0.01		0.01	±	0.01		0	±	0		0.01	±	0.01	
	June	0.03	±	0.02		0.01	±	0.01		0.02	±	0.01		0.01	±	0.01		0	±	0		0.01	±	0.01	
	July	0.03	±	0.02		0.01	±	0.01		0.02	±	0.01		0.01	±	0.01		0	±	0		0.01	±	0.01	
	August	0.03	±	0.02		0.01	±	0.01		0.02	±	0.01		0.01	±	0.01		0.01	±	0.01		0.01	±	0.01	
	September	0.03	±	0.02		0.01	±	0.01		0.02	±	0.01		0.01	±	0.01		0	±	0		0.01	±	0.01	
	October	0.03	±	0.03		0.01	±	0.01		0.02	±	0.02		0.02	±	0.01		0	±	0		0.01	±	0.01	
	November	0.03	±	0.02		0.01	±	0.01		0.02	±	0.02		0.01	±	0.01		0.01	±	0.01		0.01	±	0.01	
	December	0.03	±	0.02		0.01	±	0.01		0.02	±	0.01		0.02	±	0.01		0.01	±	0.01		0.01	±	0.01	
Vascular and unspecified dementia	January	0.04	±	0.02		0.01	±	0.01		0.02	±	0.02		0.02	±	0.01		0.01	±	0.01		0.01	±	0.01	
	February	0.04	±	0.02		0.01	±	0.01		0.02	±	0.01		0.02	±	0.02		0.01	±	0.01		0.01	±	0.01	
	March	0.04	±	0.02		0.01	±	0.01		0.02	±	0.01		0.02	±	0.01		0.01	±	0.01		0.01	±	0.01	
	April	0.03	±	0.02		0.01	±	0.01		0.02	±	0.01		0.01	±	0.01		0	±	0		0.01	±	0.01	
	May	0.03	±	0.02		0.01	±	0.01		0.02	±	0.01		0.01	±	0.01		0	±	0		0.01	±	0.01	
	June	0.03	±	0.02		0.01	±	0.01		0.02	±	0.01		0.01	±	0.01		0.01	±	0		0.01	±	0.01	
	July	0.03	±	0.02		0.01	±	0.01		0.02	±	0.01		0.02	±	0.01		0.01	±	0		0.01	±	0.01	
	August	0.03	±	0.02		0.01	±	0.01		0.02	±	0.01		0.02	±	0.01		0	±	0.01		0.01	±	0.01	
	September	0.03	±	0.02		0.01	±	0.01		0.02	±	0.01		0.02	±	0.01		0	±	0.01		0.01	±	0.01	
	October	0.04	±	0.02		0.01	±	0.01		0.02	±	0.01		0.02	±	0.01		0.01	±	0.01		0.01	±	0.01	
	November	0.04	±	0.02		0.01	±	0.01		0.03	±	0.02		0.02	±	0.01		0.01	±	0.01		0.01	±	0.01	
	December	0.04	±	0.02		0.01	±	0.01		0.02	±	0.01		0.02	±	0.01		0.01	±	0		0.01	±	0.01	

In Hokkaido prefecture, January recorded the lowest mean air temperature of the year. Among men, the number of deaths due to heart disease and cerebrovascular disease was significantly higher in January compared to other months. Pneumonia-related deaths were notably higher in January than from April to September. Deaths from accidents were significantly more frequent in January than from March to September. Renal failure deaths were significantly higher in January compared to September. For women, January saw a significant increase in deaths from heart disease compared to April through November, and from cerebrovascular disease compared to April through September. Pneumonia deaths were significantly elevated in January compared to June through August. Accidents were significantly more common in January compared to April through October, excluding May. Renal failure deaths were significantly higher in January than from May to August. No significant monthly differences were found for other causes in either sex.

In Okinawa prefecture, men experienced a higher number of deaths from heart disease in February compared to April through November, except for July. Cerebrovascular disease deaths were significantly higher in January than from April to October, excluding May and June. Pneumonia deaths were notably higher in February compared to June. Accidents were more common in January than in April, June, and November. Among women, heart disease deaths were significantly higher in January compared to May, June, October, and November. No significant monthly differences were observed for other causes in either sex.

## Discussion

The present study investigated the relationships between the number of deaths due to the 10 leading causes of death and air temperature parameters in Hokkaido and Okinawa prefectures. The findings indicated that death rates for these causes were significantly higher in Hokkaido compared to Okinawa. Furthermore, the relationship between death rates and air temperature parameters varied between the two prefectures, particularly for pneumonia, accidents, and renal failure.

Previous studies examined the relationships between the number of deaths due to various diseases, such as heart disease and cerebrovascular disease, and air temperature parameters [1-7]. Analitis et al. showed that a decrease of 1 °C in air temperature increased the rate of deaths by 1.72% for heart disease, 3.3% for lung disease, and 1.25% for cerebrovascular disease [1]. In China, 272 major cities are located in climate zones from subtropical to temperate, alpine, and cold. The mortality rates of heart disease and cerebrovascular disease were shown to be significantly higher in cold zones than in hot zones [2]. One of the major causes of heart disease and cerebrovascular disease is hypertension, and blood pressure is affected by climate and lifestyle factors [23]. Yu et al. demonstrated that when air temperature decreased by 10 °C, systolic blood pressure increased by up to 6.7 mmHg and diastolic blood pressure by up to 2.1 mmHg [5,23]. In addition, decreases in air temperature have been closely associated with increases in blood coagulation factor levels, which affect the mortality rates of heart disease and cerebrovascular disease [24]. The present results were consistent with previous findings. The number of deaths due to heart disease and cerebrovascular disease was strongly associated with air temperature parameters. No significant differences were observed in the number of deaths due to cerebrovascular disease among the months in women in Okinawa prefecture. This result may be attributed to the correlation coefficients between the number of deaths due to cerebrovascular disease and air temperature parameters being lower in women than in men in Okinawa prefecture.

The relationships between the number of deaths due to pneumonia, accidents, and renal failure and air temperature parameters differed between Hokkaido and Okinawa prefectures. The number of deaths due to pneumonia did not correlate with air temperature parameters in women in Okinawa prefecture. Jin et al. reported that the morbidity and mortality rates for coronavirus infection were higher in men than in women [25]. Immunological and genetic differences between the sexes may have contributed to these findings [26]. In addition, women are less likely to smoke or drink alcohol, and their lifestyle habits are generally better than those of men [27].

We previously reported that the number of deaths due to accidents, including asphyxiation [10], drowning [11], and falls [12], was closely related to air temperature parameters. In the present study, the number of deaths due to accidents was significantly higher in Hokkaido prefecture than in Okinawa prefecture and correlated with air temperature parameters in Hokkaido prefecture only. In Okinawa prefecture, a significant difference was noted in the number of deaths due to accidents among the months in men only. Yamamoto et al. showed that the number of deaths due to asphyxiation was markedly higher in January [10]. Deaths due to asphyxiation are common between January 1 and 3 [28] and are mainly caused by rice cakes, reflecting a characteristic of Japanese food culture [28]. The difference in air temperatures between Hokkaido and Okinawa prefectures and food culture in Japan may have contributed to these findings.

The mortality rate for acute renal failure was previously reported to be higher in winter [29]. We examined the number of deaths due to renal failure in Hokkaido and Okinawa prefectures between January 2008 and December 2016 [15], and found that it correlated with air temperature parameters in both sexes in Hokkaido prefecture, but not in women in Okinawa prefecture. In the present study, which analyzed data collected between January 2008 and December 2022, the number of deaths due to renal failure correlated with air temperature parameters, except for the lowest air temperature. The difference in the observation period may have contributed to this result.

Among the 10 leading causes of death, the number of deaths due to other diseases, i.e., malignant neoplasms, senility, aspiration pneumonia, Alzheimer’s disease, and vascular and unspecified dementia, did not correlate with air temperature parameters in the present study. Similar findings were obtained in Gifu prefecture, which is located in central Japan [30]. The number of deaths due to these causes may increase in the future due to Japan’s aging population and changing lifestyles.

These results suggest that cold weather countermeasures in Hokkaido and Okinawa should be tailored to the climatic characteristics of each region. In Hokkaido, where winter temperatures are extremely low, the risk of heart disease and cerebrovascular disease is increased. Therefore, it is essential to strengthen programs to educate residents on the proper use of heating equipment and the importance of blood pressure control. It is also recommended that heating assistance be provided to the elderly and those with preexisting medical conditions who are vulnerable to the cold, in cooperation with local communities and health centers, and that an emergency support system be established. Although Okinawa is not as cold as Hokkaido, proper temperature control during the winter and increased awareness of cold-related risks are important. Because the risks associated with cold are low, it is important to raise awareness of the risks associated with cold and implement programs to provide necessary assistance to the elderly and chronically ill. Thus, measures against cold need to be adapted to the climatic characteristics of each region.

Several limitations need to be addressed. As an ecological study, detailed individual data were not obtained. Additionally, air temperature parameters were measured at a single point in each prefecture, which may not accurately represent the overall temperatures in these areas. Furthermore, we were unable to model mortality by adjusting for variables such as time, gender, and geographic region. We also could not employ more complex study designs, like mixed designs incorporating time-trend analysis, which could have strengthened our ecological research. While our study relied on simple correlation methods to examine the relationship between air temperature and mortality in two regions, we acknowledge that a mixed design with time-trend analysis could provide deeper insights into how mortality rates vary over time and with climate changes. We plan to explore these approaches in future research to enhance our findings and gain a better understanding of temporal dynamics. Despite these limitations, the results provide a valuable dataset on the relationship between mortality due to various diseases and air temperature parameters in Japan. Further studies, including those across all 47 prefectures, are needed to confirm this relationship.

## Conclusions

In this study, we examined the relationship between mortality from the 10 leading causes of death and air temperature parameters. Our analysis revealed variations in these relationships between Hokkaido and Okinawa prefectures in Japan. Hokkaido, with its colder climate, and Okinawa, with its subtropical climate, exhibit distinct patterns in how air temperature influences mortality from these causes. This underscores the significance of accounting for regional climatic differences in public health research.
